# Temporal patterns of microglial activation in white matter following experimental mild traumatic brain injury: a systematic literature review

**DOI:** 10.1186/s40478-021-01297-1

**Published:** 2021-12-19

**Authors:** Prashanth S. Velayudhan, Nicole Schwab, Lili-Naz Hazrati, Anne L. Wheeler

**Affiliations:** 1 Department of Physiology, University of Toronto, Toronto, ON M5S 1A8 Canada; 2 Program in Neurosciences and Mental Health, The Hospital for Sick Children, Toronto, ON M5G 0A4 Canada; 3 Department of Laboratory Medicine and Pathobiology, University of Toronto, Toronto, ON M5S 1A8 Canada

**Keywords:** Microglia, Activation, White matter, Timecourses, Mild traumatic brain injury, Iba1, Corpus callosum

## Abstract

Mild traumatic brain injuries (mTBIs) are a prevalent form of injury that can result in persistent neurological impairments. Microglial activation has become increasingly recognized as a key process regulating the pathology of white matter in a wide range of brain injury and disease contexts. As white matter damage is known to be a major contributor to the impairments that follow mTBI, microglia have rightfully become a common target of investigation for the development of mTBI therapies and biomarkers. Recent work has demonstrated that the efficacy of microglial manipulation as a therapeutic intervention following injury or disease is highly time-sensitive, emphasizing the importance of advancing our understanding of the dynamics of post-mTBI microglial activation from onset to resolution. Current reporting of microglial activation in experimental studies of mTBI is non-standardized, which has limited our ability to identify concrete patterns of post-mTBI microglial activation over time. In this review, we examine preclinical studies of mTBI that report on microglial activation in white matter regions to summarize our current understanding of these patterns. Specifically, we summarize timecourses of post-mTBI microglial activation in white matter regions of the brain, identify factors that influence this activation, examine the temporal relationship between microglial activation and other post-mTBI assessments, and compare the relative sensitivities of various methods for detecting microglial activation. While the lack of replicated experimental conditions has limited the extent of conclusions that can confidently be drawn, we find that microglia are activated over a wide range of timecourses following mTBI and that microglial activation is a long-lasting outcome of mTBI that may resolve after most typical post-mTBI assessments, with the exception of those measuring oligodendrocyte lineage cell integrity. We identify several understudied parameters of post-mTBI microglial activation in white matter, such as the inclusion of female subjects. This review summarizes our current understanding of the progression of microglial activation in white matter structures following experimental mTBI and offers suggestions for important future research directions.

**Supplementary Information:**

The online version contains supplementary material available at 10.1186/s40478-021-01297-1.

## Background

Estimates have placed the annual global incidence of mild traumatic brain injury (mTBI) at over 600 cases per 100 000 people [1, 2], positioning the condition as a leading global health concern. Post-mTBI symptoms vary between people, but often include headaches, nausea, dizziness, impaired memory, poor concentration, and emotional issues such as irritability or anxiety [3]. While 80 to 90% of people experience symptoms that resolve within 7 to 10 days, the remaining 10 to 20% of individuals experience persistent symptoms that do not resolve for months or even years after injury [4].

Despite the high prevalence of mTBIs, there are no known methods to actively accelerate mTBI recovery or mitigate post-mTBI outcomes [5]. Consequently, the current state of mTBI care is restricted to advising patients to gradually return to full activity based on the progression of their symptoms [5]. The absence of effective therapeutic interventions may largely be attributed to our poor understanding of the complex and multifarious pathological sequelae of mTBIs. The heterogeneous nature of both the injuries sustained and their subsequent outcomes increases the difficulty of studying mTBIs in humans.

White matter regions of the brain, which are structures that primarily function to relay information between different brain regions, are known to be particularly vulnerable to microstructural damage in mTBIs [6, 7]. White matter damage following mTBIs has been strongly associated with the persistence of neurological deficits [8]. Though the precise mechanisms by which this damage occurs and recovers are not completely understood, some contributing processes have been identified. Diffuse axonal injury is a prevalent form of white matter damage following head trauma that is initiated by the mechanical forces of the trauma and is characterized by widespread patterns of damaged axons interspersed among intact ones, typically in midline white matter tracts such as the corpus callosum and internal capsules [6, 9]. For mild brain injuries, those mechanical forces may cause axonal cytoskeleton disruption or imbalances of intra-axonal ion concentrations that primarily lead to swelling and eventual secondary axonal disconnection. White matter damage can also occur in the form of degeneration or loss of oligodendrocyte lineage cells, which can subsequently lead to the disruption or loss of myelin. This disruption may be initiated by a variety of mechanisms after head trauma, including glutamate excitotoxicity, oxidative stress, and inflammatory consequences of activated astrocytes and microglia, reviewed in [10].

Emerging research has demonstrated a wide variety of contexts by which microglia interact with white matter during non-pathological conditions, including myelin control [11] and developmental myelination [12]. Following disease or pathology, there are several mechanisms by which microglia can function to mediate white matter damage and degeneration, including excessive post-injury myelin phagocytosis [13], inhibition of potentially assistive infiltrating macrophages [14], aberrant activation of the complement system [15], and the release of cytokines that are toxic to oligodendrocytes [16] or otherwise highly inflammatory [10, 17]. Contrasting these detrimental consequences are a variety of protective and restorative microglial functions, including debris clearance and the release of factors supporting axonal [18] and tissue regeneration [19–22]. This range of microglial functions associated with white matter injury and repair may be reflective of the many subpopulations of microglial cell types that exist during pathological conditions [23–26]. The wide range of degenerative and regenerative functions that microglia exhibit in white matter following injury position these cells as critical subjects of investigation for improving our understanding of mTBI pathologies.

Despite microglial activation being an established consequence of mTBI, the wide heterogeneity of injury parameters reported in experimental mTBI studies, including varied injury timepoints, brain regions assessed, animal models, and injury models themselves among others, create difficulties in generalizing the timecourse of microglial activation.

Detection methods of microglial activation are highly variable in regard to both technique (e.g., immunohistochemistry, electron microscopy, protein assays, transcriptional analyses, or flow cytometry) and readout (e.g., changes in microglial densities, morphologies, clustering patterns, or the detection of markers that are either specific to activated microglia or are commonly upregulated during microglial activation). These marker genes are usually associated with inflammatory microglial functions and typically do not distinguish between parenchymal microglia and infiltrating blood-derived macrophages [27]. The functions of these genes are varied, context-dependent, and still under investigation; more comprehensive summaries on what is known about them are available in other reviews [28, 29]. Though immunohistochemical quantification of ionized calcium binding adaptor molecule 1 (Iba1) and cluster of differentiation 68 (CD68) are the most commonly observed detection systems in this systematic review, there is no standardized system for choosing the marker or set of markers most appropriate for a given research question. The relative strengths and limitations of any set of markers remain poorly understood.

Understanding the timecourse and nature of microglial activation and their dependence on various injury and subject factors may be a critical step towards effectively harnessing microglia as therapeutic targets and biomarkers of injury progression.

Currently, it is common practice for experimental mTBI studies to interpret the extent of microglial activation as benchmarks for injury severity. While it does appear to be the case that more severe injuries lead to increases in the intensity and duration of microglial activation, it is not known how the usage of microglial activation as a scale of severity compares to other indicators of injury progression and outcome.

Though animal subjects commonly used for experimental mTBI such as mice have substantially less white matter than humans, many of the relevant cellular and molecular effects following injury (including microglial activation) can be reproduced in animal models [30]. In this systematic review, we examine studies of experimental mTBI that have quantified any form of microglial activation (changes in shape, size, density, count, gene expression, or cytokine production in response to injury) in white matter regions of the brain to summarize our current understanding on how subject factors, injury factors, and interventions influence microglial activation in white matter after mTBI. As well, we compare the timecourse of microglial activation to common behavioural assessments and other pathological measures to evaluate the usage of microglial activation as an indicator of injury progression and symptom resolution.

## Methods

This systematic review is built on a protocol that adheres to the Preferred Reporting Items for Systematic Review and Meta-Analysis Protocols (PRISMA-P) 2015 statement [31].

### Search strategy

On November 25th, 2020, we collected studies from Embase and PubMed using the search strategy in Table 1.

**Table 1:** Search strategy for Embase and PubMed

Search number	Query	Significance
#1	“microglia” or “microglial” or “microgliosis” or “Iba-1” or “Iba1” or “CD68” or “CD40” or “F4/80” or “CX3CR1” or “CD11b” or “CD45” or “TMEM119” or “TREM2”	Microglia
#2	“brain injury” or “brain injuries” or “brain damage” or “head injury” or “head injuries” or “head impact” or “head impacts”	Brain injuries
#3	“TBI” or “TBIs” or “mTBI” or “mTBIs”	Abbreviated head injuries
#4	“mild” or “concussion” or “concussions” or “concussive”	Mild qualifier
#5	“white matter” or “axon” or “axons” or “axonal” or “myelin” or “myelinating” or “myelination” or “demyelinating” or “demyelination” or “callosum” or “tract” or “fasciculus” or “fasciculi” or “cingulum” or “cingula” or “commissure” or “commissures” or “fornix” or “forceps” or “capsule” or “capsules” or “radiatum” or “radiata” or “semiovale” or “lemniscus” or “lemnisci” or “u-fiber” or “u-fibers”	White matter structures
#6	“diffusion” or “diffusivity” or “DTI” or “DWI” or “anisotropy” or “tractography” or “tractogram” or “tractograms”	Additional terms associated with white matter measurements
#7	#2 or #3	Pooled head injuries
#8	#5 or #6	Pooled white matter
#9	#1 and #4 and #7 and #8	Pooled microglia, mild qualifier, head injuries, and white matter

### Information sources and eligibility criteria

Articles from November 25th, 2020 and earlier mentioning microglia, white matter regions, and mTBI were searched for using PubMed and Embase. We included original, English research articles with no restrictions on date of publication or animal models used. We defined mTBI to be any traumatic brain injury that was described as mild, not described as moderate or severe, and did not induce noticeable skull fractures or brain lesions. To be included, studies needed to meet all of the following criteria: examination of experimental (preclinical) mTBI, quantification of microglial activation within at least one white matter region of the brain, statistical comparisons of microglial activation between injury groups and appropriate controls, and clear experimental design with complete reporting.

**Fig. 1:**
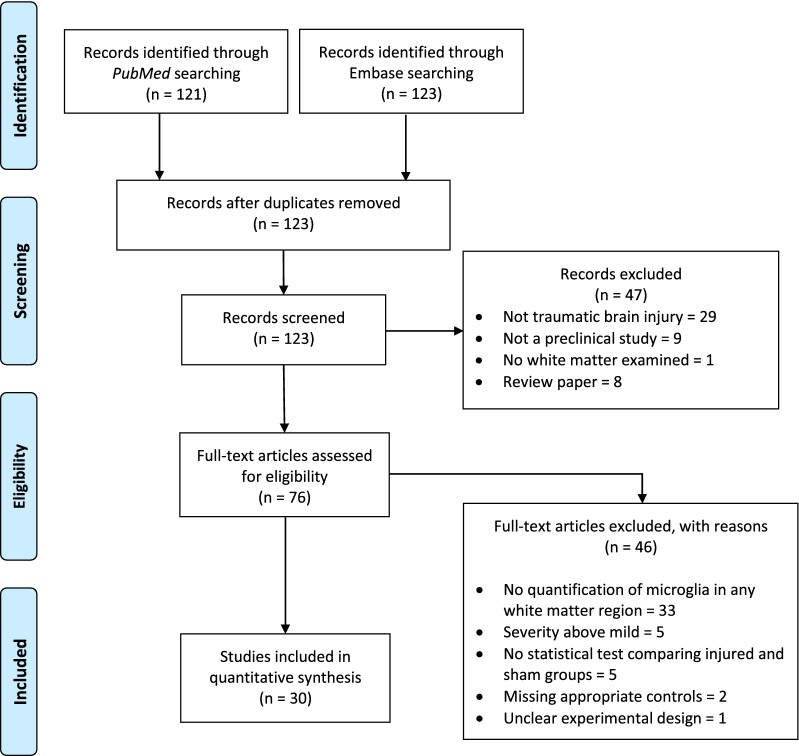
PRISMA flow diagram. Adapted from Moher D, Liberati A, Tetzlaff J, Altman DG, The PRISMA Group (2009). Preferred reporting items for systematic reviews and meta-analyses: The PRISMA Statement. PLoS Med 6(7): e1000097. https://doi.org/10.1371/journal.pmed1000097 [33]

The stages of study selection are outlined in a PRISMA flow diagram (Fig. 1). We used the risk of bias tool from the Systematic Review Centre for Laboratory animal Experimentation (SYRCLE) [32] to assess biases present in the included studies (Additional File 1). A summary of statistical methods used during the evaluation of microglial activation in the white matter for each study is provided in Additional File 2.

### Study management

Duplicate studies were removed using EndNote X8 (Clarivate Analytics, Philadelphia, PA). All remaining studies were independently screened by two of the authors for eligibility criteria using Covidence’s systematic review manager. Conflicting decisions on the inclusion status of studies were jointly discussed and resolved. Data extraction was conducted independently by two of the authors using a pre-defined Microsoft Excel form for each study. Conflicting elements of the data extraction were jointly discussed and resolved. Finished forms were merged into one spreadsheet for meta-analyses (Additional File 3).

### Data items

The complete list of data categories extracted can be found in the heading of Additional File 3. Briefly, all studies were extracted for general information (first author, year of publication, conflicts of interest), injury parameters (including number of injuries, inter-injury intervals, injury model, injury force, anesthesia, analgesia, injury location), all included assessments of impairment or pathology (behavioural tests, transcriptional analyses, protein assays, imaging metrics, histological assessments, etc.), assessment details (time between latest injury and assessment day, assessment outcome), and the sex, age, weight, and subject strain of the main experimental and control groups. When multiple experimental conditions were present, information was only extracted for conditions associated with at least one measurement of microglial activation in white matter.

### Aim and objectives

The aim of this review is to summarize our current understanding of the temporal patterns of microglial activation in white matter regions following experimental mild traumatic brain injury. To achieve this aim, we set out to address the following objectives:

1. Summarize the reported timecourses of post-mTBI microglial activation in white matter regions
2. Identify subject and injury factors that are associated with changes in those timecourses
3. Infer and compare timecourses of microglial activation across different detection methods
4. Determine whether other common post-mTBI assessments resolve before or after the resolution of microglial activation in white matter regions
5. Summarize the effects of post-mTBI interventions on microglial activation in white matter regions

### Synthesis

To summarize the timecourses of post-mTBI microglial activation in the white matter, all extracted data entries (Additional File 3) were filtered to those comparing microglial activation in a white matter region of the brain between experimental injury groups and sham controls at the same timepoints. Any entries from the same study with different microglial activation detection methods or white matter regions assessed that were otherwise identical were pooled together (Additional File 4). In cases where two entries being merged had different outcomes (i.e., one detection method showed significantly increased microglial activation in white matter relative to shams while the other showed no significant difference relative to shams), the merged entry was marked as there being a significant increase in microglial activation. This was the only way in which two entries could conflict, as there were no entries showing significant decreases of microglial activation relative to shams. We chose to merge entries to identify how long any white matter region in the brain showed any signs of microglial activation. Unmerged entries remain available in Additional File 3 for accessible integration with the results of future work. After resolving conflicts, all entries that were identical apart from the time at which microglial activation was measured were clustered into separate experimental groups. Studies were assigned numeric IDs based on the largest number of mTBIs examined by that study. Experimental subgroups within studies were distinguished with alphabetic suffixes. A plot of timelines was generated to display experimental subgroups on the y-axis and the timepoints at which they examined microglial activation on the x-axis.

To determine whether other common post-mTBI assessments resolved before or after the resolution of microglial activation in the white matter, the latest timepoint of microglial activation within an experimental group was compared to the latest timepoint of all other reported deficits of that experimental group within each study. The number of experimental groups for which each assessment resolved before, after, or inconclusively relative to the resolution of microglial activation in the white matter was tallied.

To identify subject and injury factors that were associated with differences in microglial activation in the white matter, all entries within a study that were identical apart from a single data item relating to subject factors or injury factors were tallied for whether or not the change in that factor led to an increase, decrease, or inconclusive change in the timecourse of microglial activation in the white matter. This process was repeated to track how microglial activation in the white matter varied across changing interventions and detection methods.

### Meta-biases and confidence in cumulative evidence assessments

We did not adhere to pre-defined methods of assessing potential meta-biases or relative degrees of confidence in our summarized evidence, as we did not find these assessments to be relevant to our systematic review of animal experiments.

## Results

### Included studies

**Fig. 2:**
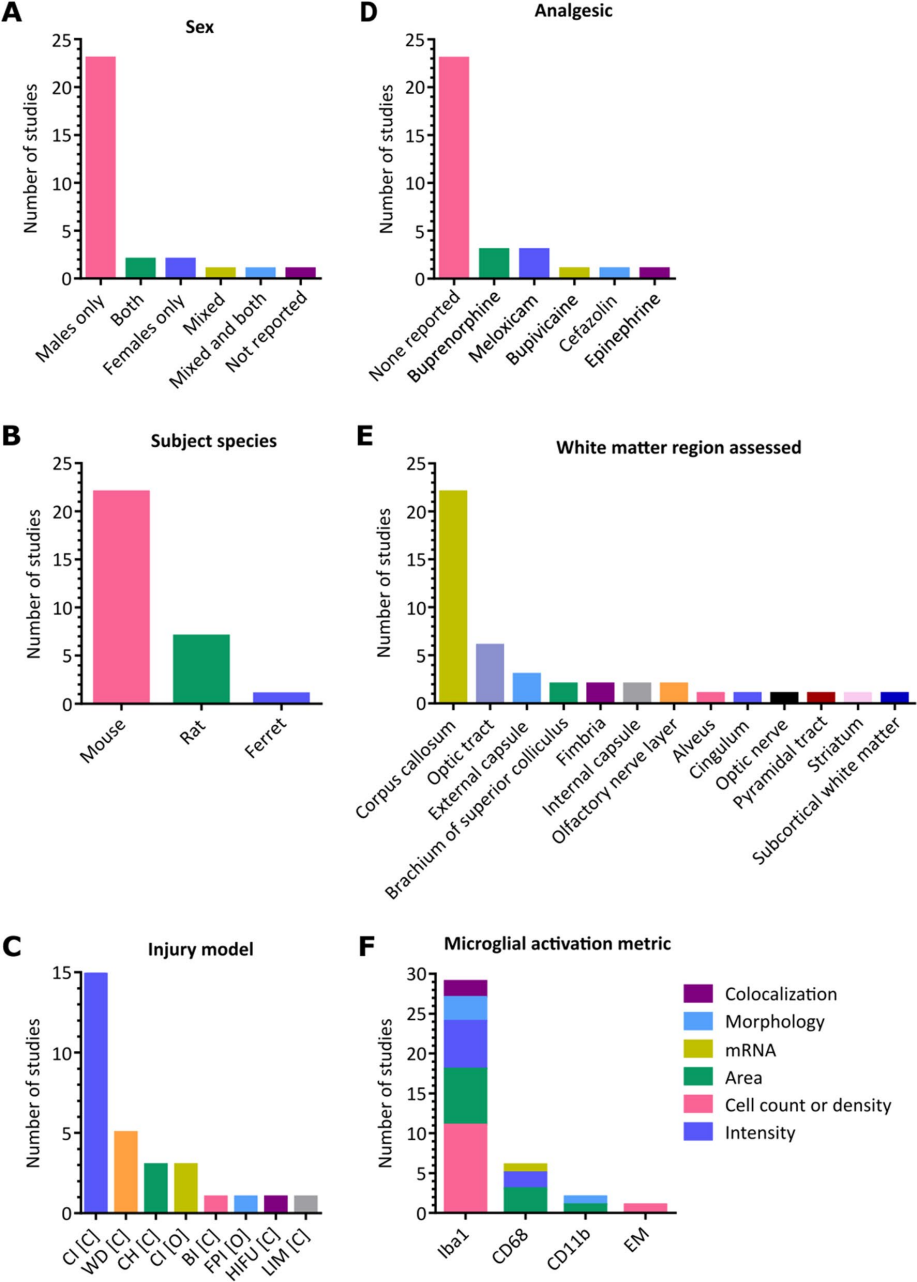
Distributions of common parameters among included studies. **A** Sexes of included subjects. Both = male and female subjects examined separately; Mixed = male and female subjects pooled together. **B** Species of included subjects. **C** Injury model used. CI = controlled piston-driven impact; WD = weight drop; CH = Closed-Head Impact Model of Engineered Rotational Acceleration (CHIMERA); BI = blast injury; FPI = fluid pulse injury; HIFU = high-intensity focused ultrasound; LIM = lateral impact model. C = closed-skull; O = open-skull. **D** Analgesia administered following mTBI procedure. **E** Regions in which microglial activation in the white matter was quantified. **F** Methods used to quantify microglial activation in the white matter. CD11b = cluster of differentiation 11b; CD68 = cluster of differentiation 68; EM = electron microscopy; H = histology; mRNA = messenger ribonucleic acid; qPCR = quantitative polymerase chain reaction

The initial search yielded 123 studies after deduplication. After screening, 30 articles were included in our quantitative synthesis. A brief summary of the parameters of the included studies is presented in Table 2. For studies containing more than one injury group or set of experimental conditions, those groups were separated into multiple rows in Table 2 and treated separately throughout the review. A summary of the distribution of major study parameters including sex, analgesic, white matter regions assessed, microglial activation detection methods, and animal models is shown in Fig. 2. An overview of each study’s contribution to Fig. 2 is available in Additional File 5.

**Table 2:** Summary of experimental groups plotted in **Fig. ****3**

ID: 1^st^ Author, Citation	Animal: Strain	Sex	Model	NOI	Other Intra-Study Differences
01a: Gatson [45]	M: C57BL/6	m	CI [C]	1	**Int.: None**
01b: Gatson [45]	M: C57BL/6	m	CI [C]	1	**Int.: Resveratrol**
02a: Goodus [37]	**M: CD1**	mix	CI [C]	1	
02b: Goodus [37]	**M: CD1-LIF**^**±**^	mix	CI [C]	1	
03a: Haber [60]	R: SD	m	CI [O]	1	
04a: Haber [61]	R: SD	m	CI [O]	1	
05a: Hernandez [101]	R: SD	m	BI [C]	1	
06a: McCabe [40]	M: C57BL/6	m	HIFU [C]	1	
07a: Namjoshi [38]	M: C57BL/6	m	CH [C]	1	
08a: Schwerin [53]	Ferret	m	CI [O]	1	
09a: Sherman [54]	M: C57BL/6	**m**	CI [C]	1	**Wt.: 37.5 g**
09b: Sherman [54]	M: C57BL/6	**m**	CI [C]	1	**Wt.: 52.5 g**
09c: Sherman [54]	M: C57BL/6	**f**	CI [C]	1	**Wt.: 52.5 g**
10a: Tu [59]	R: Wistar	f	WD [C]	1	**No MVM**
10b: Tu [59]	R: Wistar	f	WD [C]	1	**MVM**
11a: Bennett [102]	M: C57BL/6	m	CI [C]	2	
12a: Cheng [36]	**M: C57Bl/6-C3H-APP/PS1**	m	CH [C]	**2**	**Age: 24 w**
12b: Cheng [36]	**M: C57Bl/6-C3H-APP/PS1**	m	CH [C]	**2**	**Age: 54 w**
12c: Cheng [36]	**M: C57Bl/6-C3H**	m	CH [C]	**2**	**Age: 24 w**
12d: Cheng [36]	**M: C57Bl/6-C3H**	m	CH [C]	**2**	**Age: 54 w**
13b: Fidan [44]	R: SD	m	CI [C]	**1**	
13a: Fidan [44]	R: SD	m	CI [C]	**2**	
14a: Namjoshi [63]	M: C57BL/6	m	CH [C]		**Int.: None**
14b: Namjoshi [63]	M: C57BL/6	m	CH [C]	2	**Int.: Anabolic steroids**
15a: Semple [58]	M: C57BL/6	m	CI [C]	**1**	
15b: Semple [58]	M: C57BL/6	m	CI [C]	**2**	
16a: Shitaka [39]	M: C57BL/6 J	m	CI [C]	2	
17a: Fehily [43]	R: PVG	f	WD [C]	**1**	
17b: Fehily [43]	R: PVG	f	WD [C]	**2**	
17c: Fehily [43]	R: PVG	f	WD [C]	**3**	
18a: Maynard [49]	M: C57BL/6	m	WD [C]	3	
19a: Brooks [47]	R: Wistar	m	FPI [O]	4	
20a: Maynard [50]	**M: C57BL/6 J**	m	CI [C]	4	
20b: Maynard [50]	**M: B6.129 × 1-Sarm1tm1Aidi/J**	m	CI [C]	4	
21a: Bolton Hall [35]	M: C57BL/6	m	CI [C]	**1**	
21b: Bolton Hall [35]	M: C57BL/6	m	CI [C]	**5**	**III: 1**
21c: Bolton Hall [35]	M: C57BL/6	m	CI [C]	**5**	**III: 2**
22a: Eyolfson [42]	M: C57BL/6	**m**	LIM [C]	**5**	
22b: Eyolfson [42]	M: C57BL/6	**f**	LIM [C]	**5**	
22c: Eyolfson [42]	M: C57BL/6	**mix**	LIM [C]	**5**	
23a: Ferguson [48]	M: C57BL/6 J	m	CI [C]	5	
24a: Mouzon [57]	M: C57BL/6-hTau	**m**	CI [C]	5	**Age: 12 w**
24b: Mouzon [57]	M: C57BL/6-hTau	**m**	CI [C]	5	**Age: 50 w**
24c: Mouzon [57]	M: C57BL/6-hTau	**f**	CI [C]	5	**Age: 12 w**
24d: Mouzon [57]	M: C57BL/6-hTau	**f**	CI [C]	5	**Age: 50 w**
25a: Mouzon [51]	M: C57BL/6-hTau	m	CI [C]	**1**	
25b: Mouzon [51]	M: C57BL/6-hTau	m	CI [C]	**5**	
26a: Ojo [52]	M: C57BL/6	m	CI (C)	5	
27a: Yu [41]	M: C57BL/6	m	CI (C)	5	
28a: Robinson [62]	M: C57BL/6	m	WD [C]	7	
29a: Angoa-Pérez [46]	M: C57BL6/J	m	WD [C]	20	
30a: Winston [55]	M: C57BL/6	m	CI (C)	30

The majority of studies included male subjects only (23/30), whereas two studies included female subjects only. Four studies included both male and female subjects. Of those studies, two included separate male and female experimental groups (categorized as ‘Both’ in Fig. 2A), one included a single experimental group that pooled males and females together (categorized as ‘Mixed’), and one used both of the previously mentioned approaches (categorized as ‘Mixed and both’).

Most studies (22/30) used mouse models of mTBI. Some studies (7/30) used rat models, and one study used a ferret model (Fig. 2B).

Figure 2C shows the distribution of injury models across the included studies. The most commonly used injury model was some variation of closed-skull piston-driven controlled impact (CI [C]; 15 studies), followed by closed-head weight drop (WD [C]; 5 studies). The closed-head impact model of engineered rotational acceleration model (CH [C]) and open-skull piston-driven controlled impacts (CI [O]) were used in 3 studies each. The remaining injury models (blast injury (BI [C]), fluid pulse injury (FPI [O]), high-intensity focused ultrasound (HIFU [C])), and lateral impact model (LIM [C]) were present in one study each).

The usage of analgesic drugs was rarely reported. Buprenorphine and meloxicam were each used in 3 studies. Carprofen, cefazolin, and epinephrine were each used in one study each (Fig. 2D).

When assessing white matter regions for microglial activation, the corpus callosum was the most commonly sampled area (included in 22/30 studies). The optic tract was assessed in 6 studies, followed by the external capsule in 3 studies. The brachium of the superior colliculus, the fimbria, the internal capsule, and the olfactory nerve layer were included in 2 studies each. Remaining regions including the alveus, cingulum, optic nerve, pyramidal tract, striatum (structure containing both gray matter regions and white matter bundles (striatopallidal fibres / ‘pencils of Wilson’)), and subcortical white matter were assessed in one study each (Fig. 2E).

Iba1-based histology was used to detect microglial activation in 29/30 of the studies. CD68 was the next most commonly used marker, used in 6 studies. Detecting microglial activation with cluster of differentiation molecule 11B (CD11b) occurred in 2 studies. Quantification by electron microscopy was performed in one study. A breakdown of the specific quantification methods used, including colocalization-, morphology-, messenger ribonucleic acid (mRNA)-, area-, cell density-, and staining intensity-based approaches are shown in Fig. 2F.

All studies used isoflurane for anesthesia with the exception of one study, which used avertin.

### Timecourses of post-mTBI white matter microglial activation

**Fig. 3:**
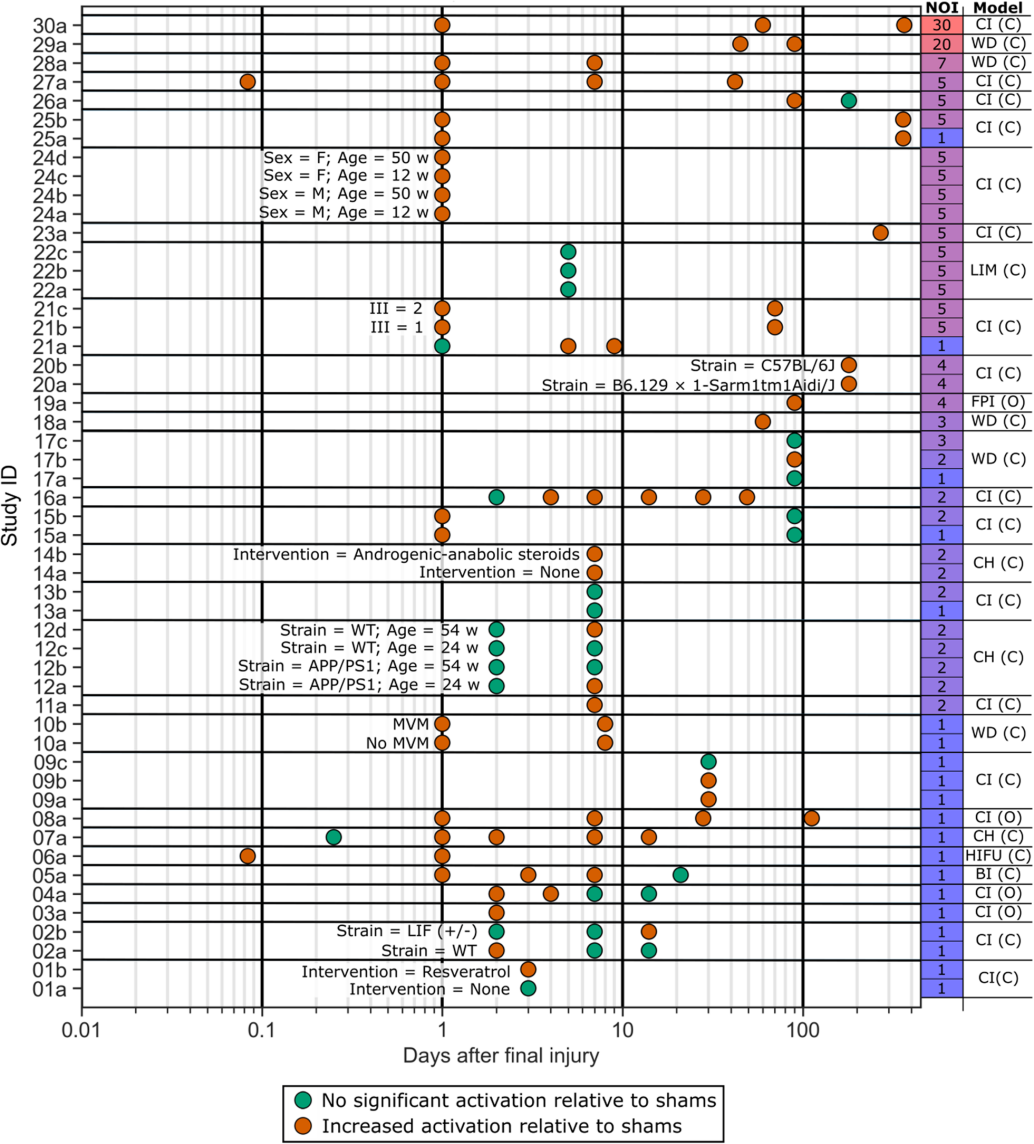
Summary of reported timecourses of microglial activation across experimental groups of included studies. The x-axis represents days since their final injury (or days since injury in the case of single mTBI studies) on a logarithmic scale. Each row along the y-axis represents one set of experimental conditions (denoted by lettering from **a** to **d** from a particular study (denoted by identification numbers 01 to 30). Studies were arranged along the y-axis by the largest number of injuries (NOI) examined by any one of their experimental groups. Experimental groups within a study were arranged by NOI if possible or arranged randomly otherwise. For studies containing multiple experimental groups that differed by a parameter other than NOI, annotations containing the differing parameters are present. The number of injuries for each experimental group is listed in a colour coded column on the righthand side of the graph. Each circle marker represents a timepoint at which microglial activation was examined. Green circle markers indicate no detected change in microglial activation in the white matter relative to shams, while orange markers indicate significantly increased microglial activation in the white matter relative to shams. Depth = impact depth of controlled impactor during mTBI; ICI = ion channel inhibitors; III = inter-injury interval; MVM = mild ventriculomegaly; NOI = number of injuries; WT = wild-type

Post-mTBI microglial activation in the white matter was defined as any property of microglia, including marker expression, morphology, and distribution pattern, that were significantly higher in white matter regions of animals receiving mTBIs when compared to otherwise identical groups receiving sham surgeries. The assessment of any form of microglial activation in the white matter and its timing post-injury is plotted in Fig. 3. Studies were assigned numeric IDs based on the largest number of mTBIs examined. Studies which examined multiple injury conditions or subject parameters were further stratified into experimental groups denoted by an alphabetic suffix. Distinct experimental groups within individual studies are plotted separately. Parameters relevant to distinguishing experimental groups within individual studies are summarized in Table 2. For the studies contributing to Fig. 3, a detailed breakdown of all parameters, including the particular detection method used to detect microglial activation, is reported in Additional File 4.

The ordering of experimental groups in Fig. 3 reveals a general trend of longer timepoints assessed for studies which examined a greater number of mTBIs. Assessments of microglial activation that showed no significant differences relative to shams appear to disproportionately occur in the lower half of Fig. 3, suggesting that a greater number of mTBIs may be associated with a longer timecourse of microglial activation.

The post-mTBI delay to microglial activation is the time taken between the injury and the onset of microglial activation. Though there is a delay to all post-mTBI microglial activation [34], only a handful of experimental studies have sampled enough early timepoints to observe it. A defined delay to post-mTBI microglial activation can be seen in groups **21a** [35], **12a** and **12d** [36], **02b** [37], **07a** [38], and **16a** [39] in the form of timecourses containing at least one early timepoint of no microglial activation (green dot in Fig. 3) preceding at least one timepoint of significantly increased microglial activation (orange dot in Fig. 3). In group **21a** [35], a delay to activation between 1 and 5 days is observed for mice receiving a single piston-driven closed-skull controlled impact. In groups **12a** and **12d** [36], the delay to activation occurs between 2 and 7 days post-final-injury (dpfi). These groups represent two closed-head impact model of engineered rotational acceleration (CHIMERA) mTBIs delivered 1 day apart to 54-week-old wild-type mice and 24-week-old APP/PS1 mice, respectively. Group **02b** [37], representing leukemia inhibitory factor (LIF) haplodeficient mice receiving a single controlled piston-driven impact, demonstrated delayed activation between 7 and 14 days post-injury. Group **07a** [38], representing male mice given a single, CHIMERA injury, shows a delay to activation between 6 h and 1 day post-injury. Finally, group **16a** [39], representing mice receiving 2 controlled piston-driven impacts separated by 1 day, show delayed activation occurring between 2 and 4 dpfi. Remaining plotted timecourses with increased microglial activation at the earliest timepoint measured suggest that those timepoints may be upper bounds for the associated groups’ delay to microglial activation. Alternatively, those groups may have sustained injuries that led to virtually no delay to microglial activation. The possibility of a microglial response occurring very shortly after injury is supported by the detection of microglial activation as early as 2 h post-final injury in groups **06a** [40] and **27a** [41], the former of which involved a single mTBI to mice. Other timecourses that show no microglial activation at any timepoint (groups **21a** [35], **12b** and **5c** [36], **22a-c** [42], **17a** and **17c** [43], **13b** and **9b** [44], and **01a** [45]) are similarly ambiguous, as they may reflect injuries which do not induce microglial activation in the white matter, injuries with delays to microglial activation in the white matter longer than the latest sampled timepoint, or injuries where microglial activation in the white matter resolved prior to the first assessment timepoint. It is important to consider that activation timecourses for experimental groups receiving multiple injuries may be misleading to directly compare with activation reported in single mTBI studies, as it is unclear if the observed activation was initiated by the final mTBI or by any of the preceding mTBIs. Accounting for the possibility that the first mTBI initiated the activation in the previously mentioned groups would lead to the following ranges of delays in microglial activation: 2 to 8 days for **12a** and **12d** [36]; 7 to 14 days for **02b** [37]; and 2 to 5 days for **16a** [39]. The findings from studies 02 [37] and 12 [36] may, however be influenced to some extent by the abnormal conditions of the experimental groups they describe. Group **12a** [36] contained APP/PS1 mice, group **12d** [36] contained aged mice, and group **02b** [37] contained LIF haplodeficient mice.

Intra-study differences are bolded for studies with multiple injury groups. M = mouse; R = rat; m = male; f = female; mix = males and females; NOI = number of injuries. WD = weight drop; CI = controlled piston-driven impact; FPI = fluid pulse injury; CH = Closed-Head Impact Model of Engineered Rotational Acceleration (CHIMERA); BI = blast injury; HIFU = high-intensity focused ultrasound; LIM = lateral impact model. (C) and (O) suffices indicate closed-skull and open-skull injury models, respectively.

Several studies report microglial activation at relatively chronic phases of the injury, ranging from 2 weeks post-final-injury to 1 year post-final-injury. These studies include groups **29a** ([46]; 20 weight drop injuries to mice spaced 1 to 3 days apart; activation lasting at least 90 dpfi), **21b** and **21c** ([35]; 5 controlled piston-driven impacts to mice spaced 1 or 2 days apart; activation lasting at least 70 dpfi), **19a** ([47]; 4 fluid pulse injuries to rats spaced 7 days apart; activation lasting at least 90 dpfi), **17a**-**c** ([43]; 2 weight drop injuries to rats spaced 1 day apart; activation lasting at least 90 dpfi), **23a** ([48]; 5 controlled piston-driven impacts to mice spaced 2 days apart; activation lasting at least 270 dpfi), **18a** ([49]; 3 weight drop injuries to mice; activation lasting at least 60 dpfi), **20a** and **20b** ([50]; 4 controlled piston-driven impacts given to mice; activation lasting at least 180 dpfi), **25a** and **25b** ([51]; 1 or 5 controlled piston-driven impacts spaced 2 days apart; activation lasting at least 360 dpfi), **26a** ([52]; 5 controlled piston-driven impacts to mice spaced 2 days apart; activation lasting at between 90 and 180 dpfi), **08a** ([53]; 1 controlled piston-driven impact to ferrets; activation lasting at least 112 dpfi), **09a** and **09b** ([54]; 1 controlled piston-driven impact to mice; activation lasting at least 30 dpfi), **16a** ([39]; 2 controlled piston-driven impacts to mice spaced 1 day apart; activation lasting at least 49 dpfi), **30a** ([55]; 30 controlled piston-driven impacts to mice spaced 1 to 3 days apart; activation lasting at least 365 dpfi), and **27a** ([41]; 5 controlled piston-driven impacts spaced 1 day apart; activation lasting at least 42 dpfi). For the groups in which the final timepoint assessed showed increased microglial activation in the white matter, it is unknown whether or not that activation will eventually resolve.

A slight positive association was observed between number of injuries and the duration of microglial activation in Fig. 3. Using a similar approach to compare timecourses between different injury models produced results that were less clear. A variation of Fig. 3 in which studies were primarily sorted by injury model and secondarily sorted by number of injuries is provided in Additional File 6. As noted in Fig. 2, the closed-head form of the controlled piston-driven impact model (CI (C)) was the primary model of choice by a substantial margin with 15 studies (29 experiments) compared to 5 studies (8 experiments) using the second most popular injury model, closed-head form of the weight drop model (WD (C)). When attempting to examine how microglial activation may compare between the two most commonly used models, CI (C) and WD (C), there was high proportion of experimental groups that showed microglial activation at the latest examined timepoint (6/8 = 75% of WD (C) and 21/29 = 72% of CI (C)). As the end of microglial activation cannot be determined in these experimental groups meaningful comparisons between the models cannot be made.

Though the timecourses presented in Fig. 3 provide some information about the potential patterns of post-mTBI microglial activation in the white matter, such as the range of delays to activation and the positive association between the number of injuries and the duration of activation, they more strikingly reveal a highly inconsistent approach to sampling post-mTBI microglial activation. Critical areas of the timecourses which appear to be particularly lacking include the temporal resolution of assessing the acute stage, for which increased assessments may lead to a better understanding of the delays to microglial activation associated with a given injury model, as well as the upper limits of assessing the chronic stage, for which later assessments may provide insights into the permanence of the neuroinflammation being induced by the original injuries.

The influences of the annotated inter-study differences in Fig. 3 on outcomes of post-mTBI microglial activation in white matter regions is examined in the following sections of the review.

### Subject and injury factors influencing post-mTBI microglial activation in the white matter

The effect of both age and Alzheimer’s status were examined in Cheng et al. [36]. Microglial activation in the white matter was measured in the optic tracts of 24-week-old and 54-week-old wild-type (WT) and APP/PS1 mice at 2 and 7 dpfi. Microglial activation in the white matter was absent on at both timepoints for the 24-week-old WT mice (ID **12c**) but only present at 7 dpfi in the 54-week-old WT mice (ID **12d**). Oddly, this age effect was reversed in APP/PS1 mice; microglial activation in the white matter was absent at both timepoints for 54-week-old APP/PS1 mice (ID **12b**) but only present at 7 dpfi in 24-week-old APP/PS1 mice (ID **12a**). The authors interpret the outcomes of the WT mice to be reflective of older animals having generally heightened microglial responses, while the outcomes of the young and old APP/PS1 respectively demonstrate a hypersensitivity of microglia around the initial time of amyloid-beta deposition and a hyposensitivity of microglia after prolonged inflammation. Further studies are required to determine if this finding occurred by chance or is indicative of a reproducible, biological phenomenon.

The effect of leukemia inhibitory factor (LIF) haplodeficiency was examined in Goodus et al. [37]. LIF is a cytokine that has been identified as playing a critical role in multiple aspects of the central nervous system’s response to injury, including neuroprotection, axonal regeneration, remyelination, and immune cell activation [56]. WT mice receiving an mTBI showed microglial activation in the corpus callosum at 2 dpfi, but no activation at 7 or 14 dpfi (ID **02a**), while LIF^±^ mice showed activation at 14 dpfi, but no activation at 2 or 7 dpfi (ID **02b**). In addition to the delayed microglial response, the LIF^±^ mice demonstrated more severe motor and sensory deficits following the mTBI than their WT counterparts. Whether the delayed response played a causative role in the exacerbated outcomes remains to be determined. The effect of the number of mTBIs on the timecourse of microglial activation in the white matter was examined in 5 studies, but conclusive answers were only found in 2 studies. In Bolton Hall et al. [35], mice receiving 5 mTBIs with an inter-injury interval of 1 day (ID **21b**) or 5 mTBIs with an inter-injury interval of 2 days (ID **21c**), but not 1 mTBI (ID **21a**), showed increased microglial activation in the corpus callosum at 1 dpfi. In Fehily et al. [43], increased microglial activation in the corpus callosum was observed at 90 dpfi in mice receiving 2 mTBI (ID **17b**), but not in mice receiving 1 mTBI (ID **17a**) or 3 mTBIs (ID **17c**). No interpretations of the increased activation observed following 2 injuries but not 3 injuries were provided.

Subject and injury factor comparisons were made between other experimental groups, but microglial activation in these cases were only assessed at timepoints that did not yield any conclusive information. This could have been due to the presence of microglial activation not being observed at any timepoint measured for both groups, persisting beyond the latest timepoint measured for both groups, or resolving between two intervals that overlapped between the two groups. In addition to the comparisons previously stated, these comparisons can be seen in Table 3.

**Table 3:** Summary of comparisons between all pairs of experimental groups that differ by only a single subject or injury factor. Sarm1-KO = sterile alpha and toll-interleukin receptor motif containing 1

Parameter	Comparable groups	Summary of comparison of microglial activation in the white matter
Age	**12a** (24 weeks) vs. **12b** (54 weeks) [36]	Increased in older group
	**12c** (24 weeks) vs. **12d** (54 weeks) [36]	Increased in younger group
	**24a** (12 weeks) vs. **24b** (50 weeks) [57]	Inconclusive comparison
	**24c** (12 weeks) vs. **24d** (50 weeks) [57]	Inconclusive comparison
Genotype	**12a** (APP/PS1) vs. **12c** (WT) [36]	Increased in APP/PS1 group
	**12b** (APP/PS1) vs. **12d** (WT) [36]	Increased in wild-type group
	**02a** (WT) vs. **02b** (LIF heterozygous) [37]	Initial increase in wild-type group, delayed increase in LIF ( ±) group
	**20a** (WT) vs. **20b** (Sarm1-KO)) [50]	Inconclusive comparison
Inter-injury interval	**21a** (1 day apart) vs. **21c** (2 days apart) [35]	Inconclusive comparison
Number of mTBIs	**21a** (1 mTBI) vs. **21b** (5 mTBIs) [35]	Increased in group with more mTBIs
	**21a** (1 mTBI) vs. **21c** (5 mTBIs) [35]	Increased in group with more mTBIs
	**17a** (1 mTBI) vs. **17b** (2 mTBIs) vs. **17c** (3 mTBIs) [43]	Highest in group with 2 mTBIs
	**13b** (1 mTBI) vs. **13a** (2 mTBIs) [44]	Inconclusive comparison
	**25a** (1 mTBI) vs. **25b** (5 mTBIs) [51]	Inconclusive comparison
	**15a** (1 mTBI) vs. **15b** (2 mTBIs) [58]	Inconclusive comparison
Sex	**22a** (males) vs. **22b** (females) vs. **22c** (mixed) [42]	Inconclusive comparison
	**24a** (males) vs. **24c** (females) [57]	Inconclusive comparison
	**24b** (males) vs **24d** (females) [57]	Inconclusive comparison
	**09b** (males) vs. **09c** (females) [54]	Inconclusive comparison
Weight	**09a** (37.5 g) vs. **09b** (52.5 g) [54]	Inconclusive comparison
Other	**10a** (no mild ventriculomegaly) vs. **10b** (mild ventriculomegaly) [59]	Inconclusive comparison

The aforementioned findings collectively suggest that post-mTBI outcomes of microglial activation in white matter regions, and possibly many other post-mTBI outcomes by extension, are complex, non-linear, and dependent on the specific combination subject details and injury conditions. That complexity provides further evidence that the approach of intentionally replicating specific experimental conditions by future studies may accelerate our understanding of the overall pathology of mTBIs at a much faster rate than the approach of testing novel experimental conditions and attempting to integrate the subsequent findings with the existing literature.

### Comparisons of methods for the detection and analysis of microglial activation

Though the detection of microglial activation is a common assessment following experimental mTBI, a considerable variety of methods were employed across the studies. There were 5 included studies which quantified microglial activation in the white matter in the same injury models using more than one approach. The summaries of these inter-approach comparisons are provided in Table 4.

**Table 4:** Summary of comparisons of methods for the detection and analysis of microglial activation

Microglial activation detection method	Study	Summary of comparison
CD11b + area vs. CD11b + morphology	Yu et al. [41]	CD11b + morphology showed higher sensitivity
CD68 + area vs. CD68 qPCR	Haber et al. [61]	CD68 + area showed higher sensitivity
CD68 + area vs. Iba1 + area	Haber et al. [60]	CD68 + area showed higher sensitivity
	Haber et al. [61]	Iba1 + area showed higher sensitivity
CD68 + intensity vs. Iba1 + intensity	Robinson et al. [62]	Inconclusive comparison
Iba1 + area vs. CD68 qPCR	Haber et al. [61]	Iba1 + area showed higher sensitivity
Iba1 + area vs. Iba1 + clustering	Schwerin et al. [53]	Iba1 + clustering showed higher sensitivity

In Haber et al. [60], the corpus callosum of mice given mTBIs showed increased immunoreactivity for CD68, but not Iba1, at 2 dpfi. In Haber et al. [61], increased Iba1 immunoreactivity in the corpus callosum was observed on 2 and 4 dpfi, while increased CD68 immunoreactivity was only observed at 2 days post-injury. In Robinson et al. [62], both Iba1 and CD68 were observed to have increased immunoreactivity in the fimbria of mTBI mice at 1 and 7 dpfi, which did not provide any conclusive evidence towards their orders of occurrence. Future work employing higher temporal resolution is required to determine the relative ordering of Iba1- and CD68-based activation.

In Schwerin et al. [53], the subcortical white matter of ferrets given one mTBI were measured for percentage area labeled of Iba1^+^ cells and for the number of Iba1^+^ cell clusters at 1, 7, 28, and 112 dpfi. Increased percentage area labeled was observed at 7 and 112 dpfi (the only reported instance of bimodal microglial activation across all included studies), while increased cell clustering was observed at 28 and 112 dpfi. In Yu et al. [41], CD11b^+^ cells in the corpus callosum of mice given 5 mTBIs were quantified by percentage area labeled and by the number of cells exhibiting non-resting morphologies. Activation by morphology and activation by area labeled were detected at 1, 7, and 42 dpfi, but only morphological analysis revealed significant activation at 2 h post injury.

These findings highlight both the complexity of microglial dynamics following mTBI as well as the impact of choice in analytical method on the determination of microglial activation. Understanding whether the relative sensitivities of the different approaches are injury-specific or have a consistent pattern that has yet to be identified may require a more formal investigation than can be provided by a meta-analysis of the current literature.

### Persistence of microglial activation relative to other deficits and injury markers

**Fig. 4:**
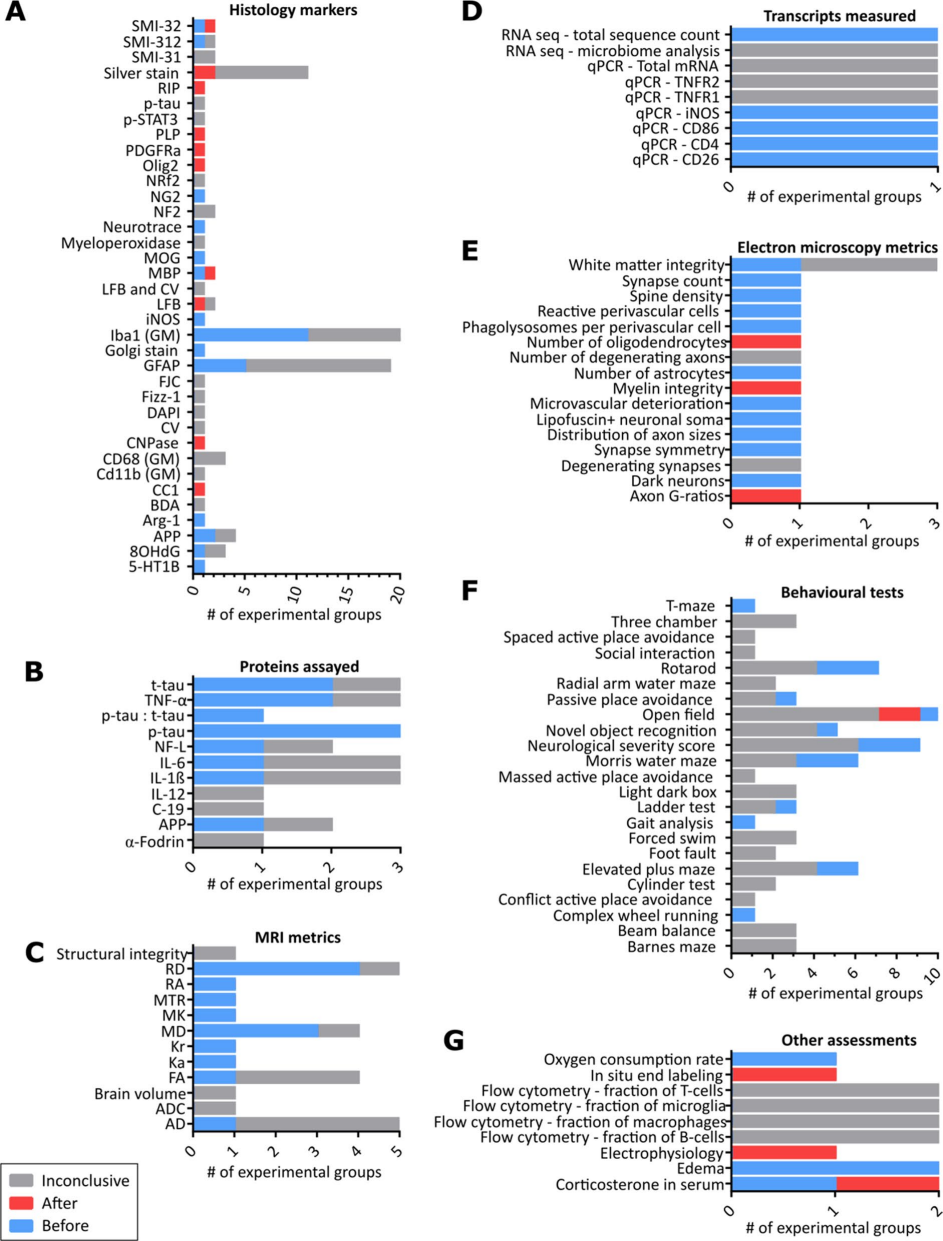
Contextualizing microglial activation in white matter relative to other post-mTBI assessments. ‘Before’ and ‘after’ tallies refer to when an assessment resolved relative to the resolution of microglial activation. Instances where limited temporal range or resolution of the assessment obscured the relative orders of resolution were tallied as ‘Inconclusive’. Specific assessment descriptions are provided in Additional File 7. 5-HT1B = 5-hydroxytryptamine receptor 1B; 8-OHdG = 8-hydroxy-2’-deoxyguanosine; AD = axial diffusivity; ADC = apparent diffusion coefficient; APP = amyloid precursor protein; Arg-1 = arginase-1; BDA = biotinylated dextran amine; CD11b = cluster of differentiation molecule 11b; CD206 = cluster of differentiation 206; CD40 = cluster of differentiation 40; CD68 = cluster of differentiation 68; CD86 = cluster of differentiation 86; CNPase = 2’,3’-Cyclic-nucleotide 3’-phosphodiesterase; CV = cresyl violet; DAPI = 4’,6-diamidino-2-phenylindole; FA = fractional anisotropy; FJC = Fluoro-Jade C; GFAP = glial fibrillary acidic protein; IL-1β = interleukin 1 beta; IL-12 = interleukin 12; IL-6 = interleukin 6; iNOS = inducible nitric oxide synthase; Ka = axial kurtosis; Kr = radial kurtosis; LFB = luxol fast blue; MBP = myelin basic protein; MD = mean diffusivity; MK = mean kurtosis; MOG = myelin oligodendrocyte glycoprotein; mRNA = messenger ribonucleic acid; MTR = magnetization transfer ratio; NF200 = neurofilament protein; NF-L = neurofilament light protein; NG2 = neural/glial antigen 2; Nrf2 = NF-E2 DNA binding protein; Olig2 = oligodendrocyte transcription factor; PDGFRα = Platelet-derived growth factor receptor α; PLP = proteolipid protein 1; p-STAT3 = phospho-signal transducer and activator of transcription 3; p-tau = phospho-tau; qPCR = quantitative polymerase chain reaction; RA = relative anisotropy; RD = radial kurtosis; RNA = ribonucleic acid; TNF-α = tumor necrosis factor alpha; TNFR1 = tumor necrosis factor receptor 1; TNFR2 = tumor necrosis factor receptor 2; t-tau = total tau

We tallied the number of times all reported behavioural and pathological metrics of mTBI resolved before, after, or at inconclusive timepoints relative to the resolution of microglial activation in white matter regions (abbreviated as B:A:I; Fig. 4). The complete breakdown of the experimental groups contributing to Fig. 4 are provided in the “Assessment tallies” tab of Additional File 7. Brief descriptions of the included assessments are provided in the “Summary of assessments” tab of Additional File 7. Generally, studies made use of behavioural, histological, protein measurement, and mRNA measurement techniques to measure outcomes in learning, memory, motor coordination, axonal and neuronal degeneration, microglial and astrocytic activation, and regulation of inflammatory cytokines. Tallies from studies which only assessed microglial activation at immediate/early-acute timepoints (less than 7 days) were considered inconclusive to avoid misinterpreting cases in which microglial activation was yet to occur for cases in which it had rapidly resolved or would never begin. These studies included Eyolfson et al. ([42]; Study ID **2**; activation measured at 5 dpfi) and Gatson et al. ([45]; Study ID **10**; activation measured at 3 dpfi). Additionally, experimental groups with additional factors that may not be reflective of the general recovery process following mTBI were excluded. The excluded experimental groups were **12a** and **12b** (Cheng et al. [36]; animals were APP/PS1 mutants), **01b** (Gatson et al. [45]; mice were treated with resveratrol), **02b** (Goodus et al. [37]; mice had LIF^±^ genotype), **20b** (Maynard et al. [50]; animals were Sarm1-KO mutants), **18** and **19** (Mouzon et al. [57] and Mouzon et al. [51]; animals were hTau mutants), **14b** (Namjoshi et al. [63]; animals were treated with androgenic–anabolic steroids), and **10b** (Tu et al, [59]; animals had mild ventriculomegaly).

Histological measures (Fig. 4) were the most commonly reported outcomes of mTBI among the included studies. Many studies suggested that both Iba1^+^ gray matter activation (Fig. 4A; B:A:I: = 11:0:13; experimental groups **21b**-**c**, **17b**, **05a**, **06a**, **08a**, **15a**-**b**, **09a**, **09c** and **16a** contribute to the before tallies) and brain-wide glial fibrillary acidic protein (GFAP)^+^ astrocytic activation (B:A:I: = 5:0:14; experimental groups **17b**, **07a**, **15a-b**, **27a** contribute to the before tallies) persist for less time than microglial activation in the white matter.

A large proportion of the markers associated with oligodendrocyte lineage cells showed abnormal levels persisting after the resolution of microglial activation in the white matter. The oligodendrocyte lineage markers in Fig. 4A include anti-adenomatous polyposis coli clone CC1 (B:A:I: = 0:1:0), 2’,3’-Cyclic-nucleotide 3’-phosphodiesterase (CNPase; B:A:I: = 0:1:0), myelin basic protein (MBP; B:A:I: = 1:1:0), myelin oligodendrocyte glycoprotein (MOG; B:A:I: = 1:0:0), neural/glial antigen 2 (NG2; B:A:I: = 1:0:0), oligodendrocyte transcription factor (Olig2; B:A:I: = 0:1:0), platelet-derived growth factor receptor α (PDGFRα; B:A:I: = 0:1:0), proteolipid protein 1 (PLP; B:A:I: = 0:1:0), and Rip (B:A:I: = 0:1:0). These assessments primarily came from experimental groups **02a** (male and female mice receiving a single, closed-skull, controlled piston-driven impacts) and **04a** (male mice receiving a single, open-skull, controlled piston-driven impact).

All protein levels (Fig. 4B), magnetic resonance imaging metrics (Fig. 4C), and mRNA levels (Fig. 4D) measured in post-mTBI brain across the included studies had either returned to sham levels before the resolution of microglial activation in the white matter or had persisted at least as long as its latest detection. Targets of protein and mRNA analyses were sparsely distributed across studies and predominantly focused on markers of microglia, macrophages, and inflammation. No transcripts were measured by more than one study.

Of the electron microscopy metrics (Fig. 4E), the number of oligodendrocytes (B:A:I: = 0:1:0), myelin integrity (B:A:I: = 0:1:0), and the average G-ratio of axons within the corpus callosum (B:A:I: = 0:1:0) were the only measures to have any occurrences of resolving later than microglial activation in the white matter. Though there is only a single tally for each of these measures, these findings support the possibility of prolonged post-mTBI oligodendrocyte lineage cell impairment following seen in Fig. 4A.

Most behavioural deficits following mTBI resolved prior to the resolution of microglial activation in the white matter or at inconclusive timepoints (Fig. 4F). The only exception was the open field test (B:A:I: = 3:2:7), which may better temporally correlate with microglial activation in the white matter than the other tallied tests. The two ‘after’ tallies for the open field test came from the experimental groups **15a** and **15b** [58], which included male mice receiving one or two closed-skull, controlled piston-driven impacts, respectively.

Remaining assessments (Fig. 4G) included measures of oxygen consumption rate, in situ end labeling (ISEL), flow cytometry counts of various immune cells in the brain parenchyma, electrophysiological recordings (conduction speeds of the corpus callosum), brain swelling, and serum corticosterone levels. ISEL (B:A:I: = 0:1:0), electrophysiology (B:A:I: = 0:1:0), and serum corticosterone (B:A:I: = 1:1:1) were the only metrics of these to have any occurrences of resolving later than microglial activation in the white matter. Similar to most other assessments tallied, more work employing these techniques is required before their order of resolution relative to the resolution of microglial activation in the white matter can be confidently determined.

These findings appear to collectively demonstrate that while microglial activation in white matter regions may be one of the most persistent effects of mTBI, it is likely outlasted by changes in the structure and expression of oligodendrocyte lineage cells.

### Effects of interventions on microglial activation in the white matter and post-mTBI outcomes

Two of the included studies examined the effects of therapeutic interventions on microglial activation in the white matter and other post-mTBI outcomes.

In Gatson et al., 2013 [45], mice were treated with placebos (ID **01a**) or 100 mg/kg resveratrol (ID **01b**) at 5 min and 12 h following mTBI. Resveratrol (3,4’,5-trihydroxystilbene) is a plant-derived phenol and phytoalexin that has been suggested to have beneficial anti-inflammatory and immunomodulatory effects [64]. Unlike mice receiving placebos, the mice that were treated with resveratrol post-mTBI displayed no microglial activation in the corpus callosum, and no increases in hippocampal levels of the pro-inflammatory cytokines IL-6 or IL-12 at 3 dpfi. Whether the intervention delayed, prevented, or accelerated the resolution of microglial activation is unknown. Additionally, the absence of behavioural testing and measures of brain tissue integrity at later timepoints make it unclear if the altered microglial response was associated with a better post-mTBI outcome.

In Namjoshi et al. [63], male mice were treated with either a vehicle (group **14a**) or a combination of androgenic–anabolic steroids (AAS; group **14b**) including testosterone, nandrolone, and 17α-methyltestosterone for 8 weeks starting at the age of 8 weeks. Mice received 2 CHIMERA mTBIs spaced 1 day apart at the 7th week of their AAS or vehicle treatment, and assessments were carried out over the following week. Compared to vehicle-treated mice, the mice treated with the AAS combination showed no changes in behavioural performance but did have increased axonal damage and magnitude of microglial activation. However, as both the vehicle and AAS-treated groups showed increased microglial activation relative to shams at the only time of tissue collection (7 days dpfi), there was no conclusive difference in the timecourse of microglial activation observed between the two groups.

These studies indicate that the usage of interventions may be highly effective in altering the persistence of microglial activation in white matter regions following mTBI. Further studies will be required to determine how the direction of manipulating microglial activation and the timing of those manipulations interact to influence neurological recovery and brain repair.

## Discussion

In this systematic review, we summarized the current state of literature regarding the timecourse of microglial activation following experimental mTBI. Despite the known significance of microglia in regulating white matter damage and repair following injury, there is insufficient information in the literature to confidently identify any robust patterns of post-mTBI microglial activation in the white matter. Post-mTBI microglial activation in the white matter reported in the included studies revealed a wide range of possible timecourses that remain challenging to explain given the limited number of studies for any particular set of experimental parameters. A single injury could not trigger any noticeable increase in microglial activation in some cases (e.g., ID **13b** [44]) but initiated microglial activation persisting for over a year in others (e.g., ID **25a** [51]). As well, the possibility of multimodal activation, as seen in a subgroup of Schwerin et al., 2018 [53] may be commonly overlooked by low sampling frequencies following mTBI.

One unknown relationship raised by the comparisons of the timelines observed in Fig. 3 and Figure S1 of Additional File 6 is the possibility that different injury models may produce different activation durations. In this review, any further investigation into this question was hindered by the small number of studies using injury models other than the CI (C) model as well as the high proportion of experimental groups for which the end of microglial activation was never determined.

Initial steps have been made towards understanding how different subject and injury factors can influence the course of mTBI. Among the studies included in this review, microglial activation in the white matter has been demonstrably altered by varying age [36], genotype [36, 37], number of injuries [35, 43], and therapeutic intervention [45, 63]. No conclusive determinations were made on the influence of inter-injury interval or subject sex on microglial activation in the white matter. The general trend of excluding female subjects from experimental mTBI research is problematic, particularly given the substantial body of evidence suggesting that males and female experience different outcomes following head injury [65–69]. There is an urgent need to increase research on the sex differences of the pathological mechanisms underlying mTBIs. The effects of other common mTBI parameters, such as injury location or the dosage and choice of anesthesia and analgesia were not examined by any of the included studies. Understanding how these parameters influence the course of post-mTBI recovery may allow for improved mTBI treatment plans in a clinical setting. The effect of inter-injury interval, which was only investigated by one study in this review ([35] – ID **21b** vs. **21c**) is especially important for groups with a high risk of repeat mTBI, including military personnel, children, the elderly, survivors of domestic violence, and athletes, as post-mTBI returns to full activity are currently entirely dependent on symptom resolution rather than any biological marker of resolution in brain vulnerability.

The limitations of symptom-based guidelines for mTBI patients returning to high-risk activity are emphasized by the majority of detectable behavioural deficits in experimental groups resolving prior to the end of microglial activation in the white matter. A patient with persisting neuroinflammation that sustains a subsequent mTBI may experience longer lasting and more serious impairment as a consequence [70]. Though the behavioural methods tallied in this review are not perfectly representative of the post-mTBI deficits that humans experience, the possibility of incomplete brain recovery persisting beyond the apparent resolution of symptoms for any patient of mTBI may be a valid generalization.

Though the mechanistic investigations of microglia are not typically conducted in studies related to mTBI, works from other neuropathological contexts have made progress in identifying specific details by which microglia can influence behavioural performance, protein and transcriptional levels, and microstructural brain changes during development, homeostasis, and pathology. Some examples include the capability of microglia to shape neural circuitry during development using the complement signaling pathway [71], that microglia can repair myelin following injury in a neuronal activity-dependent mechanism [72], that overexpression of the eukaryotic translation initiation factor 4E (eIF4E) in microglia causes autism-like behaviours in male mice only [73], that microglial activity detectable by positron emission tomography is a strong correlate of white matter structural integrity in patients of multiple sclerosis [74], and that microglia can contribute to Alzheimer’s Disease pathology by regulating tau phosphorylation [75].

While the observation that microglial activation persisted beyond the resolution of most other mTBI assessments in Fig. 4 may appear to indicate that these cells do not causally contribute to those impairments and deficits, substantial evidence from previous work demonstrating that microglia play critical roles in both the injury and repair components of neuropathological conditions (the concept of ‘good’ and ‘bad’ microglia in mTBI are reviewed in detail elsewhere [76]) suggest that this is not necessarily be true. It is instead more likely that activated microglia contributed to the development and resolution of some, if not all, of the assessments included in Fig. 4. For assessments that hypothetically progress completely independently of any microglial influence, the value of identifying their order of resolution relative to microglial activation in white matter regions is primarily restricted to identifying which assessments may be the most effective mTBI biomarkers.

Oligodendrocyte lineage marker disruption was found to persist after the resolution of microglial activation. Given the substantial body of evidence suggesting that white matter impairment is one of the strongest correlates of impairment following mTBI [6–8, 77], we interpret this finding as support for the possibility that microglia contribute to prolonged impairment following mTBI by mediating lasting damage to white matter structures of the brain. This damage may be caused during periods of overactivation but persist well after microglia have returned to homeostatic levels. Though microglia also function to repair white matter pathology following injury, the incomplete recovery of oligodendrocyte lineage cells to a homeostatic transcriptional signature could be indicative of those repair processes ending prematurely. This persistent white matter damage may be too subtle to detect through traditional experimental behavioural tests but may compound after additional injuries into more serious impairment. In the clinical context, this may be analogous to slight alterations in cognition or behaviour that are not easily observed by current techniques in symptom assessment.

The usage of more sophisticated methods of characterizing microglial activation is of high importance. Though the broader field of microglial research has moved on to investigating microglial activation as a multi-dimensional phenomenon, the mTBI studies featured in this review have all focused on relatively simple activation models. Some included studies have moved beyond the exclusive usage of markers like Iba1 or CD68 and have instead focused on the M1/M2 schema, which proposes a spectrum of activation along two major microglial phenotypes: “classical” activation of pro-inflammatory (M1) microglia and “alternative” activation of anti-inflammatory (M2) microglia. This model has since been considered an oversimplified system that may hinder our understanding of microglial biology more than it helps [78], resulting in recent decline in usage.

Currently, microglia are considered to execute their vast range of functions by adopting a similarly vast range of subtypes and reaction states, typically characterized through single-cell RNA sequencing. A recent review by Stratoulias et al. [79] provides a highly detailed overview of several major microglial subtypes reported as of 2019 alongside many of the key points of debate regarding the definition and identification of novel subtypes.

Disease-associated microglia (DAM) [80], a population with downregulated C-X3-C motif chemokine receptor 1 (Cx3cr1), cluster of differentiation 33 (CD33), and transmembrane protein 119 (Tmem119), were found to significantly slow the progression of a mouse model of AD.

Inflammatory-responsive microglia, characterized by upregulated levels of galectin 3 (Lgals3), cystatin F (Cst7), C–C motif chemokine ligand 4 (Ccl4), C–C motif chemokine ligand 3 (Ccl3), and interleukin 1 beta (Il1b) [81], were identified as potential drivers of age-related neuroinflammation.

Activated response microglia (ARMs), characterized by overexpression of MHC type II, Dickkopf-related protein 2 (Dkk2), hematopoietic growth factor inducible neurokinin-1 type (Gpnmb), and secreted phosphoprotein 1 (Spp1) [82], were identified as potential mediators of both age-, sex-, and genetic-based heightened risk for AD.

One microglial phenotype that is especially relevant to the subject of white matter damage is the integrin alpha X (CD11c) + microglia that were potentially first identified in a 1994 multiple sclerosis pathology study by Ulvestad et al. [83]. These cells are now recognized to play major roles in the regulation of myelin across developmental, homeostatic, and pathological contexts (described in detail by a 2020 review from Benmamar-Badel et al. [84]).

Though any of these phenotypes may contribute to mTBI pathology, there were notably no studies in this review which attempted to deeply characterize the microglial diversity associated with their preclinical models of mTBI. Future studies which take care to consider the role of microglia through the lens of highly diverse subtypes and activation states may greatly improve our understanding of how findings from different preclinical models of brain injury or other neuropathological conditions relate to each other and to the clinical mTBI setting.

In the context of detecting neuroinflammation in vivo as a biomarker of post-mTBI recovery, the current gold standard is the positron emission tomography (PET) of translocator protein (TSPO), which becomes highly upregulated in microglia following activation [85]. However, the invasiveness and exposure to radiation required for TSPO PET imaging may limit its usage in clinical contexts as a biomarker for mild brain injuries. Advancements in magnetic resonance imaging (MRI) techniques may eventually alternatively permit the accurate, whole-brain characterization of morphologically activated microglia in live subjects in a substantially less invasive way [86, 87].

Studies that pair these sophisticated techniques of detecting microglial activation with experimental designs that prioritize increased temporal resolution may begin to identify practical post-mTBI therapeutic windows to target microglia. The value in understanding the timing of microglial activation can be clearly seen in such studies as Willis et al. [88], in which microglial depletion and subsequent repopulation with a drug at the moment of injury had great therapeutic value, but the same treatment administered with a delay after injury had no beneficial effect. Similarly, in Brody et al. [89], constant microglial depletion starting 7 days prior to the injury and ending 21 days after the injury showed no therapeutic value.

In addition to improving the temporal resolution with which microglial activation is assessed, future studies should take increased care during the selection of markers used to identify activated microglia. Many markers commonly used to detect activated microglia are found on resting microglia as well. Some markers thought to be specific to activated forms of microglia include major histocompatibility complex II (MHC II) [90], translocator protein (TSPO) [91], restin-like alpha (FIZZ1) [92], and matrix metalloproteinase-9 (MMP9) [93]. Alternative approaches to using activation-specific markers include assessing the relative ratios of common microglial markers to identify activated signatures or to characterize activated morphologies among cells identified through individual common microglial markers [29].

Another concern associated with marker selection is the lack of discrimination between innate microglial cells of the central nervous system and peripheral macrophages which are capable of infiltrating the brain parenchyma upon injury [94, 95]. Markers which cannot distinguish between peripheral macrophages and microglia when used alone include CX3CR1, Iba1, cluster of differentiation 45 (CD45), epidermal growth factor-like module-containing mucin-like hormone receptor-like 1 (F4/80), and CD11b [96]. Some markers that are currently considered to be specific to microglia include transmembrane protein (TMEM119), P2Y purinoceptor 12 (P2RY12), and Sal-like protein 1 (SALL1) [97].

The work in Brody et al. [89] also highlights how the absence of microglial activation itself may not be unconditionally therapeutic; it is likely the specific types of activation blocked and the timing of those blocks that are critical in providing reversing and minimizing the damage that can follow an mTBI. In the context of interventions, we are still far from understanding mechanistic links between compounds affecting microglial activation and how they improve the outcomes of mTBIs. This gap must be addressed in order to develop clinically promising candidate therapies that are specific, selective, and effective.

Ultimately, the discordance of experimental paradigms, the lack of temporal range and resolution of data collection, and the limited numbers of markers employed for detecting microglial activation have greatly hindered our understanding of the underlying temporal patterns of microglial activation in white matter following experimental mTBI. Consequently, a cohesive model describing the timecourse of microglial activation following mTBI remains a goal for future syntheses. For future studies to accelerate our understanding of these patterns, we strongly encourage researchers using experimental mTBI models to consider replicating injury parameters from previously published studies in addition to increasing the range and resolution of microglial activation measurements in white matter regions.

Though a variety of these parameters may be beneficial for identifying broad patterns of microglial activation that are more likely to have translational value, it is clear that the current field of the role of microglia in experimental mTBI is spread thin. Improving the normalization of data collection in future studies will be an invaluable approach to improving our understanding of the role of microglia and the mechanisms underlying mTBI as a whole at a faster pace. One of the challenges of such normalization that is likely responsible for the discordance of the current literature is that the selection of precise injury model parameters, assessment timepoints, and markers is a largely arbitrary process. We do not have specific recommendations for any of those parameters, but for future experimental studies examining microglia in the context of post-mTBI white matter damage, we suggest three experimental design choices which may contribute to a more harmonized body of literature from which meaningful interpretations can more easily be drawn from: 1) when establishing a new model of mTBI or using a model for which the post-mTBI delay to microglial activation or duration of microglial activation are unknown, examine microglial activation in as many early acute (~ 1–7 days [98–100]) and chronic (> 14–60 days [98–100]) timepoints as possible; 2) when selecting experimental parameters, assessment techniques, and assessment timepoints for a new study, consider matching or including the choices from previous studies; 3) when characterizing microglial activation, use specific markers of microglial activation, use multiple markers, and use single cell ribonucleic acid (RNA)-sequencing or other rigorous transcriptional characterization approaches when possible.

### Limitations

As a consequence of including studies that may have been statistically underpowered (see Additional File 2), this review has a risk of misinterpreting instances where one group has a lower magnitude but longer lasting period of microglial activation relative to another group as a situation in which that second group has the longer lasting activation. This review also has a risk of mischaracterizing the relative order of the resolution of different post-mTBI changes which used assessment methods of different sensitivities.

The analyses performed in this review relied on multiple assumptions and simplifications that limit the strength of their conclusions. When summarizing timecourses of microglial activation in the white matter, assessments within experimental groups involving different white matter regions or detection methods of microglial activation were pooled together. As well, the individual timecourses were not separated by detection method or white matter region. These simplifications allowed the timecourses observed across all studies to be directly compared but may lead to misleading interpretations when comparing timecourses that were generated from white matter regions or detection methods that have substantially different sensitivities to microglial activation in the white matter. Additionally, the merging of entries may have concealed evidence of differences in specific microglial subtypes generated by different experimental conditions. At this time, we believe that the lack of harmony across the included studies mitigates much of the potential consequence of this decision by precluding the possibility of any confident speculations of specific microglial subtypes from being generated.

All post-mTBI outcomes, including microglial activation, were treated as having unimodal timecourses of activation. This could lead to mischaracterizing timecourses of microglial activation in the white matter or of other assessments as having resolved prematurely. No analyses in this study accounted for differing impact locations between studies.

Another limitation of this review is that we did not restrict our search or inclusion criteria to only those studies which used detection methods specific to microglial activation. As the field of experimental mTBI moves towards more sophisticated and rigorous approaches to characterizing microglial activation, sufficient literature will become available with which such a review can be conducted.

During the preparation of the results in this review, there was no difference in weighting for each of the experimental groups in a multi-group study (e.g., the 4 groups present in [57]) or a single experimental group from a single-group study (e.g., the 1 group present in [46]). Consequently, our findings have a bias towards multi-group studies, where a greater number of experimental groups results in a greater degree of bias.

### Conclusion

The summaries presented in this systematic review highlight several areas of post-mTBI microglial activation in the white matter research that require additional investigation. Some remaining questions about microglial activation are presented in Box 1. Increased efforts to replicate experimental conditions and verify existing data are needed to confidently interpret the timecourse of microglial activation following mTBI. Future studies applying higher temporal and transcriptional resolution will be critical towards understanding the natural role of microglia in the course of post-mTBI resolution and novel strategies to manipulate microglia in ways that can facilitate complete and improved post-mTBI recovery.

Box 1 - Outstanding questions

1. What is the delay time for microglia activation to occur post-mTBI and what factors influence this?
2. What is the relative order of the resolution of microglial activation across different white matter regions and how does that associate with the relative vulnerability of those regions to mTBI?
3. How does the location of the mTBI influence microglial activation throughout the brain?
4. How does sex influence the duration of post-mTBI microglial activation in the white matter?

## Supplementary Information

**Additional file 1**: SYRCLE Risk of Bias Tool. Risk of bias assessment for included studies.

**Additional file 2**: Statistical tests used to evaluate microglial activation. File containing the statistical tests used by each study to evaluate microglial activation as well as a summary of if and how power analyses were included.

**Additional file 3**: Full extractions. Complete table of data items extracted from included studies.

**Additional file 4**: Pooled extractions for microglial activation timecourses. Data items relevant to timecourses of microglial activation in white matter regions.

**Additional file 5**: Summary of major study parameters. File containing the parameters of each study that contributed to Figure 2.

**Additional file 6**: Supplementary Figure 1: Timecourses arranged by injury model. A variation of Figure 3 in which timecourses are arranged by injury model rather than number of injuries.

**Additional file 7**: Tallies to contextualize microglial activation. Tab 1: Evaluations of the relative order of the resolution of microglial activation in the white matter compared to the resolution of other included assessments for included studies. Tab 2: Descriptions of included assessments.

## Data Availability

All data in this systematic review was obtained from the included articles. All data generated or analysed during this systematic review are included in this published article and its supplementary information files.

## Abbreviations

5-HT1B: 5-Hydroxytryptamine receptor 1B
8-OHdG: 8-Hydroxy-2’-deoxyguanosine
AAS: Androgenic–anabolic steroids
APP: Amyloid precursor protein
Arg-1: Arginase-1
ARMs: Activated response microglia
B:A:I: Ratio of tallies for mTBI assessments resolving before, after, or at inconclusive timepoints relative to the resolution of microglial activation in white matter regions
BDA: Biotinylated dextran amine
BI: Blast injury
[C]: Suffix indicating closed-skull injury
Ccl3: C–C motif chemokine ligand 3
Ccl4: C–C motif chemokine ligand 4
CD11c: Integrin alpha X
CD206: Cluster of differentiation 206
CD33: Cluster of differentiation 33
CD40: Cluster of differentiation 40
CD45: Cluster of differentiation 45
CD68: Cluster of differentiation 68
CH: Closed-head impact model of engineered rotational acceleration
CHIMERA: Closed-head impact model of engineered rotational acceleration
CI: Piston-driven controlled impact
CNPase: 2’,3’-Cyclic-nucleotide 3’-phosphodiesterase
Cst7: Cystatin F
CV: Cresyl violet
CX3CR1: C-X3-C motif chemokine receptor 1
DAM: Disease-associated microglia
DAPI: 4’,6-Diamidino-2-phenylindole
Dkk2: Dickkopf-related protein 2
dpfi: Days post-final-injury
eIF4E: Eukaryotic translation initiation factor 4E
EM: Electron microscopy
F4/80: Epidermal growth factor-like module-containing mucin-like hormone receptor-like 1
FJC: Fluoro-Jade C
FIZZ1: Restin-like alpha
FPI: Fluid pulse injury
GFAP: Glial fibrillary acidic protein
Gpnmb: Hematopoietic growth factor inducible neurokinin-1 type
HIFU: High-intensity focused ultrasound
Iba1: Ionized calcium binding adaptor molecule 1
ICI: Ion channel inhibitors
IL-12: Interleukin 12
IL-1β: Interleukin 1 beta
IL-6: Interleukin 6
iNOS: Inducible nitric oxide synthase
ISEL: In situ end labeling
LFB: Luxol fast blue
Lgals3: Galectin 3
LIF: Leukemia inhibitory factor
LIM: Lateral impact model
MBP: Myelin basic protein
MHC II: Major histocompatibility complex II
MMP9: Matrix metalloproteinase-9
MOG: Myelin oligodendrocyte glycoprotein
MRI: Magnetic resonance imaging
mRNA: Messenger ribonucleic acid
mTBI: Mild traumatic brain injury
NF200: Neurofilament protein 200
NF-L: Neurofilament light protein
NG2: Neural/glial antigen 2
Nrf2: Neurofilament 2
NOI: Number of injuries
[O]: Suffix indicating open-skull injury
Olig2: Oligodendrocyte transcription factor
P2RY12: P2Y purinoceptor 12
PDGFRα: Platelet-derived growth factor receptor α
PET: Positron emission tomography
PRISMA-P: Preferred reporting items for systematic review and meta-analysis protocols
PLP: Proteolipid protein 1
p-STAT3: Phospho-signal transducer and activator of transcription 3
p-tau: Phospho-tau
qPCR: Quantitative polymerase chain reaction
RNA: Ribonucleic acid
SALL1: Sal-like protein 1
Sarm1-KO: Sterile alpha and toll-interleukin receptor motif containing 1
Spp1: Secreted phosphoprotein 1
SYRCLE: Systematic review centre for laboratory animal experimentation
TMEM119: Transmembrane protein 119
TNF-α: Tumor necrosis factor alpha
TNFR1: Tumor necrosis factor receptor 1
TNFR2: Tumor necrosis factor receptor 2
TREM2: Triggering receptor expressed on myeloid cells 2
TSPO: Translocator protein
t-tau: Total-tau
WD: Weight drop
WT: Wild-type
