# Supplementary material for: Temporal patterns of microglial activation in white matter following experimental mild traumatic brain injury: a systematic literature review
Source: Acta Neuropathol Commun. 2021 Dec 19;9:197. doi: 10.1186/s40478-021-01297-1 (PMC8684664; doi:10.1186/s40478-021-01297-1)
Supplement: Supplementary file 1 — Additional file 1: SYRCLE Risk of Bias Tool. Risk of bias assessment for included studies. [file 40478_2021_1297_MOESM1_ESM.docx]

Risk of bias assessment for included studies. Y = yes; N = no; U = unclear. Questions: Q1) Was the allocation sequence adequately generated and applied? Q2) Were the groups similar at baseline or were they adjusted for confounders in the analysis? Q3) Was the allocation adequately concealed? Q4) Were the animals randomly housed during the experiment? Q5) Were the caregivers and/or investigators blinded from knowledge which intervention each animal received during the experiment? Q6) Were animals selected at random for outcome assessment? Q7) Was the outcome assessor blinded? Q8) Were incomplete outcome data adequately addressed? Q9) Are reports of the study free of selective outcome reporting? Q10) Was the study apparently free of other problems that could result in high risk of bias? Taken from SYRCLE’s risk of bias tool (Hooijmans et al., 2014)*.*

| Study | SYRCLE’s risk of bias tool question | | | | | | | | | |
| --- | --- | --- | --- | --- | --- | --- | --- | --- | --- | --- |
|  | Q1 | Q2 | Q3 | Q4 | Q5 | Q6 | Q7 | Q8 | Q9 | Q10 |
| Angoa-Perez et al., 2020 | Y | Y | U | U | Y | Y | U | Y | Y | Y |
| Bennett et al., 2012 | U | U | U | U | U | U | Y | Y | Y | U |
| Bolton Hall et al., 2016 | Y | Y | U | U | U | Y | Y | Y | Y | Y |
| Brooks et al., 2017 | Y | U | U | U | U | U | Y | Y | Y | Y |
| Cheng et al., 2018 | U | U | U | U | U | Y | U | Y | Y | Y |
| Eyolfson et al., 2020 | U | Y | U | U | Y | Y | Y | Y | Y | Y |
| Fehily et al., 2019 | Y | Y | U | U | Y | Y | Y | Y | Y | Y |
| Ferguson et al., 2017 | U | U | U | U | Y | Y | Y | N | N | N |
| Fidan et al., 2016 | U | U | U | U | U | Y | U | Y | Y | Y |
| Gatson et al., 2013 | U | U | U | U | U | U | U | Y | Y | Y |
| Goodus et al., 2016 | U | U | U | U | U | Y | U | Y | Y | Y |
| Haber et al., 2013 | U | U | U | U | U | U | U | N | N | N |
| Haber et al., 2018 | Y | Y | U | U | U | U | U | Y | Y | Y |
| Hernandez et al., 2018 | U | Y | U | U | Y | Y | Y | Y | Y | Y |
| Maynard et al., 2019 | U | Y | U | U | U | U | U | Y | Y | Y |
| Maynard et al., 2020 | U | Y | U | U | U | Y | U | Y | Y | Y |
| McCabe et al., 2014 | U | Y | U | U | U | U | U | U | N | Y |
| Mouzon et al., 2018 | Y | Y | U | U | Y | Y | Y | Y | Y | Y |
| Mouzon et al., 2019 | Y | Y | U | U | Y | Y | Y | U | Y | Y |
| Namjoshi et al., 2016 | Y | Y | U | U | U | Y | Y | Y | Y | Y |
| Namjoshi et al., 2017 | U | Y | U | U | U | Y | U | Y | Y | Y |
| Ojo et al., 2015 | U | U | U | U | U | Y | U | Y | U | Y |
| Robinson et al., 2017 | Y | Y | Y | U | Y | Y | Y | Y | N | Y |
| Schwerin et al., 2018 | U | U | U | U | Y | Y | Y | Y | Y | Y |
| Semple et al., 2016 | U | Y | Y | U | Y | Y | Y | Y | Y | Y |
| Sherman et al., 2016 | U | U | U | U | U | U | U | Y | Y | N |
| Shitaka et al., 2011 | U | Y | U | U | Y | Y | Y | N | N | Y |
| Tu et al., 2017 | Y | Y | U | U | Y | Y | Y | Y | Y | Y |
| Winston et al., 2016 | U | U | U | U | U | Y | Y | Y | Y | Y |
| Yu et al., 2017 | U | U | U | U | Y | Y | Y | Y | Y | Y |
