# Supplementary material for: Temporal patterns of microglial activation in white matter following experimental mild traumatic brain injury: a systematic literature review
Source: Acta Neuropathol Commun. 2021 Dec 19;9:197. doi: 10.1186/s40478-021-01297-1 (PMC8684664; doi:10.1186/s40478-021-01297-1)
Supplement: Supplementary file 6 — Additional file 6: Supplementary Figure 1: Timecourses arranged by injury model. A variation of Figure 3 in which timecourses are arranged by injury model rather than number of injuries. [file 40478_2021_1297_MOESM6_ESM.docx]

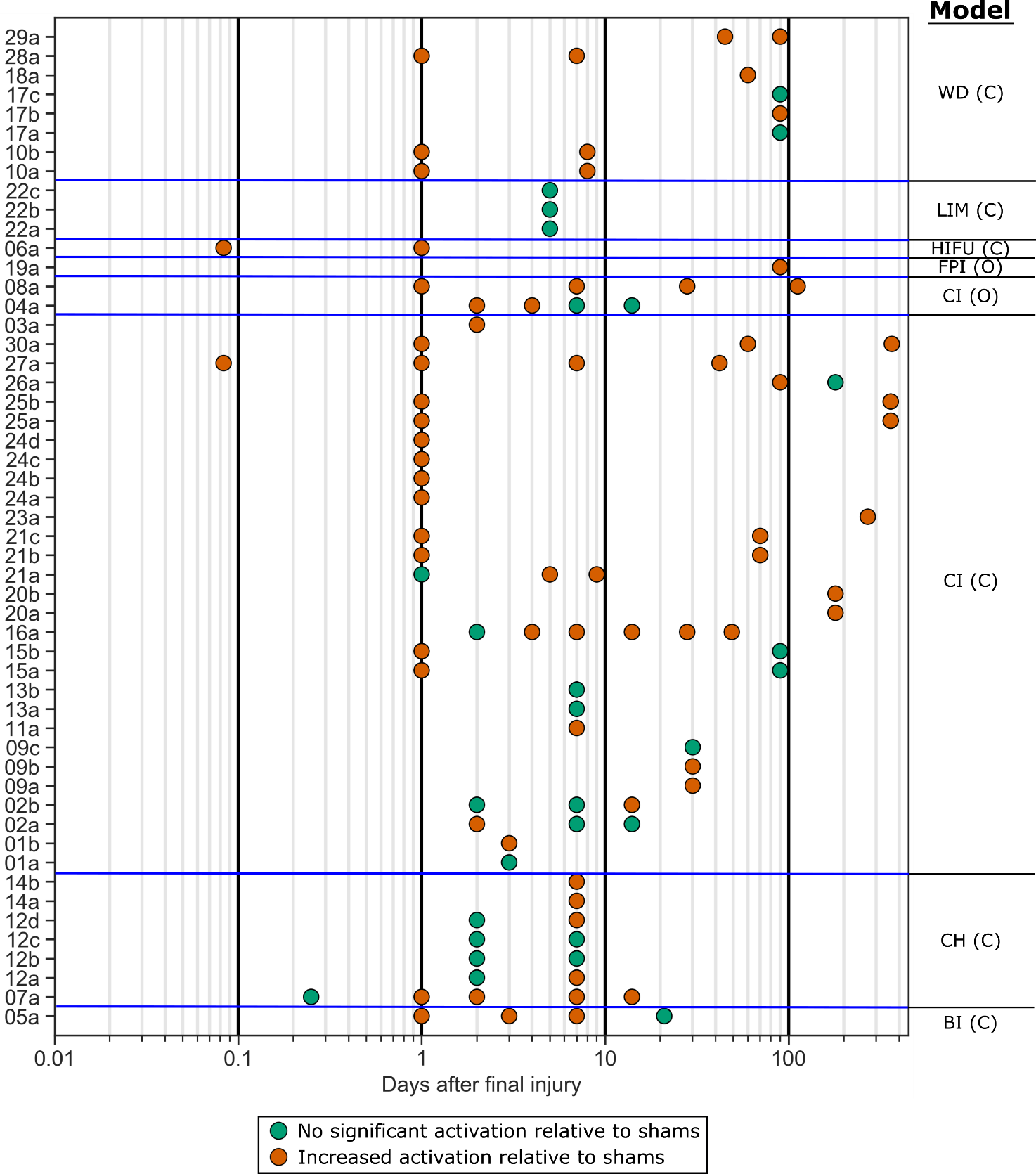


Figure S1 – Variation of Figure 3 (summary of reported timecourses of microglial activation) for which studies were arranged along the y-axis by injury model. Studies using the same injury model were arranged by the largest number of injuries within that study. BI = blast injury; CH = Closed-Head Impact Model of Engineered Rotational Acceleration (CHIMERA); CI = controlled piston-driven impact; FPI = fluid pulse injury; HIFU = high-intensity focused ultrasound; LIM = lateral impact model; WD = weight drop. (C) and (O) suffices indicate closed-skull and open-skull injury models, respectively.
